# Association of Insulin Resistance with Glucose and Lipid Metabolism: Ethnic Heterogeneity in Far Western China

**DOI:** 10.1155/2016/3825037

**Published:** 2016-12-22

**Authors:** Yi-Zhong Yan, Ru-Lin Ma, Jing-Yu Zhang, Jia He, Jiao-Long Ma, Hong-Rui Pang, La-Ti Mu, Yu-Song Ding, Heng Guo, Mei Zhang, Jia-Ming Liu, Dong-Sheng Rui, Kui Wang, Shu-Xia Guo

**Affiliations:** ^1^Department of Preventive Medicine, University of Shihezi, Shihezi 832000, China; ^2^Department of Pathology and Key Laboratory of Xinjiang Endemic and Ethnic Diseases (Ministry of Education), Shihezi University School of Medicine, Shihezi 832000, China

## Abstract

*Objective*. To study the relationships between IR and glucose and lipid metabolism in far western China and these relationships' ethnic heterogeneity.* Methods*. From the baseline survey, 419 Uygur cases, 331 Kazak cases, and 220 Han cases were randomly selected, resulting in a total of 970 cases for study. FINS concentration was measured by radioimmunoassay.* Results*. (1) In the Kazak population, IR was correlated with hyperglycemia; high levels of TC, TG, and LDL-C; and low levels of HDL-C and abdominal obesity (all *P* < 0.05). (2) In the Uygur population, the influence of IR on hyperglycemia and abdominal obesity was the greatest. In the Kazak population, IR was associated with hyperglycemia most closely. In the Han population, IR may have had an impact on the incidence of low HDL-C levels. (3) After adjusting for sex, age, smoking status, and alcohol consumption, IR was still associated with anomalies in the metabolism of the Uygur, Kazak, and Han populations.* Conclusion*. IR was involved in the process of glucose and lipid metabolism, and its degree of involvement differed among the ethnicities studied. We could consider reducing the occurrence of abnormal glucose and lipid metabolism by controlling IR and aiming to reduce the prevalence of metabolic syndrome and related diseases.

## 1. Introduction

In recent years, the prevalence of diabetes, dyslipidemia, and obesity has still been a growing trend, affecting human health seriously. Studies have found that metabolic diseases often occur simultaneously in an individual and manifest as metabolic syndrome (MS), which can greatly increase a person's risk of cardiovascular disease [1, 2]. However, although the reasons for this clustering are still unclear, a large number of studies have agreed that insulin resistance (IR) is the root cause and has a common pathophysiological origin [3–5]. IR is most closely related to glucose metabolism [6]. As a predictor of diabetes, IR is an important precursor to and warning sign for type 2 diabetes. Obesity, and especially abdominal obesity, is a key factor leading to the development of IR. Current epidemiological evidence has demonstrated a strong correlation between obesity and IR among patients with MS [7]. However, the relationship between abnormal lipid metabolism and IR remains controversial, as the common changes related to IR-related dysfunction are high TG and low HDL-C levels but the link between IR and both TC and LDL-C is weaker [8, 9].

China is a multiethnic country and there are more than 10 ethnic groups in Xinjiang Uyghur Autonomous Region, where Uygur, Kazak, and Han are three large inhabitant ethnic groups. However, there are many differences among these ethnic groups, such as religion, culture, lifestyle, diet, and genetic background. Concerning the diet of Uygur and Kazak population, especially, their primary foods are wheat, beef, mutton, and dairy products containing high fat,but they consume less fruits and vegetables than Han. Due to limited resources in public health and poor transportation, there have not been serious investigations to analyze local public health including MS and related diseases; we need to pay more attention to their health and we need to improve health.

We have found that the prevalence of metabolic diseases such as obesity, hyperglycemia, and lipid metabolism disorders differs among the Uygur, Kazak, and Han populations. Numerous studies have confirmed that IR is the common thread of these metabolic diseases; thus, among the ethnic groups in Xinjiang, we examined the relationships between these diseases and IR. This study aimed to analyze the relationships between IR and glucose and lipid metabolism as well as these relationships' ethnic heterogeneity. Furthermore, we aimed to identify information relevant to preventing MS and related diseases, which may contribute to establishing appropriate preventive public health policies for different ethnic groups.

## 2. Subjects and Methods

*(1) Subjects*. Our survey was conducted from 2009 to 2012 in Yili, Kashi, Shihezi, Tacheng, and Changji prefectures. This survey collected information about MS from residents (≥18 years old). On this basis, we randomly selected 419 Uygur cases, 331 Kazak cases, and 220 Han cases, resulting in a total of 970 cases for study.

*(2) Diagnostic Criteria and Related Definitions*. (1) Dyslipidemia is TC ≥ 6.22 mmol/L as hypercholesterolemia; TG ≥ 2.26 mmol/L as hypertriglyceridemia; LDL-C ≥ 4.14 mmol/L as high low-density lipoprotein cholesterol; HDL-C < 1.04 mmol/L as low high-density lipoprotein cholesterol. Any of these lipids is abnormal that is judged as dyslipidemia [10]. (2) Hyperglycemia is FBG ≥ 6.1 mmol/L and (or) diagnosed with diabetes and treatment [11]. (3) Abdominal obesity is waist circumference for men ≥ 85 cm and women ≥ 80 cm [12]. The homeostasis model assessment of insulin resistance (HOMA-IR) index was defined as follows: [fasting insulin (mU/L) × fasting glucose (in mM)]/22.5. The Chinese Diabetes Society (CDS) states that IR can be estimated using this formula in epidemiological or clinical studies, and the upper quartile of the subjects was the split point [13].

*(3) Laboratory Tests*. (1) TC, TG, LDL-C, HDL-C, and fasting glucose levels were assessed by a biochemical autoanalyzer (Olympus AU 2700, Olympus Diagnostics, Hamburg, Germany) in a clinical laboratory. (2) FINS was determined by radioimmunoassay with kit purchased from Beijing Atomic-Tech Co. Ltd. (Beijing, China).

*(4) Statistical Analysis*. All of the analyses were performed using the SPSS statistical package for Windows (version 19.0). Continuously and normally distributed variables were analyzed using variance analysis, and the results are presented as the mean ± standard deviation (M ± SD). Variables with a skewed distribution were analyzed using the Mann–Whitney *U*-test, and the results are expressed as the median (upper quartile, lower quartile) (*M*(*Q*_*u*_, *Q*_*L*_)). Factors were analyzed using multivariate logistic regression analysis.

## 3. Results

### 3.1. Description of the General Situation

There was a total of 970 cases, including 419 cases of Uygur (43.2%), 331 cases of Kazak (34.1%), and 220 cases of Han (22.7%). The nationalities were divided into two groups (IR and Non-IR) according to the upper quartile of IR (Uygur: 1.17 mmol/L, Kazak: 0.97 mmol/L, and Han: 0.31 mmol/L), and average age and gender were not significantly different (*P* > 0.05 for each comparison); TG, TC, HDL-C, LDL-C, FBG, and WC were different (*P* < 0.05 for each comparison) (Table 1).

### 3.2. The Relationships of IR and Glucose and Lipid Metabolism

#### 3.2.1. Univariate Logistic Regression Analysis

The results of univariate logistic regression analysis using hyperglycemia, low HDL-C, high TG, high LDL-C, high TC, and abdominal obesity as dependent variables and IR as the independent variable showed that when stratifying by IR quartiles the rates of detection of hyperglycemia showed an increasing trend with an increased IR incidence in the Uygur, Kazak, and Han populations. The Kazak and Han groups showed statistical significance in the fourth quintile, whereas the Uygur group had the highest odds ratio (OR) value in the third quintile. The detection of low HDL-C levels showed an increasing trend with an increased incidence of IR, with the Uygur and Kazak groups showing statistical significance in the fourth quintile, whereas the Han group had the highest OR value in the second quintile. The detection of high TG levels showed an increasing trend with an increased incidence of IR, with the Uygur, Kazak, and Han groups showing statistical significance in the fourth quintile. The detection of high LDL-C levels showed an increasing trend with an increased incidence of IR, with the Uygur and Kazak groups showing statistical significance in the fourth quintile, whereas the Han population showed statistical significance in none of the quintiles. The detection of high TC levels showed an increasing trend with an increased incidence of IR, with the Kazak and Han groups showing statistical significance in the fourth quintile, whereas the Uygur population showed statistical significance in none of the quintiles. The detection of abdominal obesity showed an increasing trend with an increased incidence of IR, with the Kazak and Han groups showing statistical significance in the fourth quintile, whereas the Uygur group had the highest OR value in the third quintile (Tables 2–4).

#### 3.2.2. Multivariate Logistic Regression Analysis

To exclude the influence of confounding factors (age, gender, smoking, and drinking), we had used multivariate Logistic regression with hyperglycemia, low HDL-C, high TG, high LDL-C, high TC, and abdominal obesity as the dependent variable and IR as the independent variable; the results showed that, in Uygur, high TC was not included by the equation, but smoking entered, and OR value of abdominal obesity was highest. IR of Kazak was still associated with all of the indicators, and OR value of hyperglycemia was highest. However, there was no relationship between IR and high LDL-C for Han, but smoking entered the equation, and OR value of low HDL-C was highest (Table 5–7).

## 4. Discussion

IR can exist before the development of diabetes and cardiovascular disease [14]. A prospective study found significant correlations among hyperinsulinemia, low HDL-C, and diabetes [15]. In addition, IR showed certain differences among different ethnic groups. In the present study, after stratifying the subjects according to the IR quartiles, we found that in the fourth quintile the IR of the Kazak population was highest and that of the Han population was lowest, which indicated that IR may be a more serious risk factor in the Kazak population; this finding was consistent with other reports [16, 17].

As early as 1988, studies found that obesity and IR have a causal relationship, as the accumulation of excessive adipose tissue can induce IR [18]. The characteristics of IR caused by obesity are the inhibition of hepatic glucose output and the promotion of glucose uptake by muscle and adipose tissue. Shao et al. found that 11*β*-HSD1 inhibition can exert a potential benefit in terms of reducing obesity and lowering IR by modulating the insulin signaling pathway and adipocytokine production [19]. However, the prevalence of obesity has increased on an annual basis. According to one survey, the prevalence of adult obesity (male 35.5%, female 35.8%) in the US in 2010 had reached 30.0% in advance [20] of the levels projected for 2015 [21]. In our study, among the Uygur, Kazak, and Han populations, we found that IR was associated with abdominal obesity; this relationship was observed at the third quintile of IR in the Uygur population, while the Kazak and Han groups showed this association in the fourth quintile. These data suggest that lower levels of IR could influence the Uygur population. In addition, the OR was highest in the Han population, which indicated that the correlation between IR and abdominal obesity was strongest in the Han population.

IR is most closely related to glucose metabolism and is a predictor of diabetes. Type 2 diabetes is always accompanied by IR, and hyperglycemia may appear 10 years earlier than other clinical symptoms of diabetes [22, 23]. The UKPDS study found that at the time of diagnosis with type 2 diabetes patients' islet *β* cell function was only 50% of that in normal humans and that this function gradually declined if the diabetes continued its course [24]. Cusi [25] found that the IR was most obvious in patients with diabetes after comparing between patients with normal glucose tolerance and patients with impaired glucose tolerance. These data were consistent with the clinical findings, which also showed that IR and diabetes were closely related. Our study found that the rate of detection of hyperglycemia increased with an increased incidence of IR, which suggests that IR is associated with hyperglycemia. Interestingly, the Kazak and Han populations showed statistical significance in the fourth quintile, whereas the Uygur group had the highest OR value, at 13.490, in the third quintile, which showed that the impact of IR on hyperglycemia was greatest in the Uygur population.

Related studies have reported that the most common factors associated with IR were high TG levels and low HDL-C levels but that the relationships between IR and both LDL-C and TC were weaker [9] . The present study was consistent, with the conclusion that among the Uygur, Kazak, and Han populations, overall, IR was closely related to high TG levels and low HDL-C levels, whereas there was no relationship between IR and either high LDL-C in the Han population or high TC in the Uygur population. Bao et al. [26] reported that in all types of dyslipidemia, both high TG levels and high TG-induced hyperlipidemia were associated with the presence of other IR-related lipid abnormalities as well as with an increasing degree of aggravated dyslipidemia. Certain studies have shown that TG is an independent variable that is involved in serum lipid-induced IR and that is positively correlated with high TG-induced IR lipid marker levels [27]. In the present study, the high TG detection rates in the Uygur, Kazak, and Han ethnic groups were stratified by increasing levels of IR, and this relationship displayed significance in the fourth quintile. One of the hallmarks of IR is a reduced plasma concentration of HDL-C, suggesting that IR and HDL-C are closely related [28]. In this study, lower rates of IR in the Han population showed a correlation with HDL-C, with an OR of 10.800. However, in the other two ethnic groups, this correlation was significant in the fourth quintile, which showed a more obvious correlation between IR and HDL-C than in the Han population.

In short, the degree of correlation between IR and various metabolic disruptions was inconsistent; one explanation for this finding was ethnic heterogeneity. In the Uygur population, IR affected hyperglycemia and abdominal obesity less and was more closely associated with hyperglycemia, without any correlation with TC. In the Kazak population, the relationships of IR with various metabolic dysfunctions occurred in the group in the fourth quintile and were most closely related to hyperglycemia. Finally, in the Han population, the influence of IR on low HDL-C was most obvious, with the highest OR value, whereas high LDL-C levels did not show any correlation with IR.

## 5. Conclusion

In summary, IR is the common pathophysiological cause of a variety of metabolic abnormalities. In the present study, among the Uygur, Kazak, and Han populations, there were significant differences, but because this was only a cross-sectional study we could not identify the real reason for these differences. However, this study highlights the relevance of the relationship between IR and various metabolic disorders; based on this, we can observe and analyze larger groups and possibly perform experimental studies to eventually identify fundamental differences. These potential studies may provide a scientific foundation for formulating measures and strategies for the prevention and treatment of metabolic diseases that are suitable for different ethnic groups, with the aim of improving the quality of human life.

## Figures and Tables

**Table 1:** General situation.

Ethnic	Group	Male/female	Age (years)	TG (mmol/L)	TC (mmol/L)	HDL-C (mmol/L)	LDL-C (mmol/L)	FPG (mmol/L)	WC (cm)
Uygur	IR	153/162	41.61 ± 12.29	1.48 ± 0.96	4.54 ± 1.10	1.14 ± 0.29	2.51 ± 0.76	4.53 ± 0.79	86.69 ± 11.77
	Non-IR	46/58	41.82 ± 11.81	1.16 ± 0.72	4.40 ± 1.24	1.25 ± 0.27	2.44 ± 0.67	4.03 ± 0.64	83.49 ± 10.19
	*P*	0.897	0.653	<0.001	0.012	0.005	0.023	<0.001	0.008
Kazak	IR	110/137	45.83 ± 11.40	1.38 ± 1.37	4.52 ± 1.04	1.37 ± 0.39	2.34 ± 0.74	4.94 ± 1.50	89.08 ± 11.69
	Non-IR	36/48	46.33 ± 9.95	1.10 ± 0.61	4.39 ± 1.00	1.41 ± 0.38	2.27 ± 0.73	4.26 ± 0.84	86.64 ± 10.86
	*P*	0.472	0.800	<0.001	<0.001	<0.001	0.032	0.017	0.028
Han	IR	66/99	49.44 ± 11.23	1.89 ± 1.41	4.81 ± 1.12	1.41 ± 0.37	2.82 ± 0.80	5.40 ± 2.46	88.22 ± 10.00
	Non-IR	32/23	49.62 ± 9.38	1.40 ± 0.68	4.65 ± 0.96	1.52 ± 0.26	2.70 ± 0.63	4.87 ± 0.75	84.80 ± 9.50
	*P*	0.502	0.823	<0.001	<0.001	0.037	0.029	<0.001	<0.001

Notes: TG = triglyceride, HDL-C = high-density lipoprotein cholesterol, FPG = fasting plasma glucose, LDL-C = low-density lipoprotein cholesterol, TC = total cholesterol, and WC = waist circumference.

**Table 2:** The detection rates of glucose and lipid metabolism by IR level in Uygur.

Metabolism	Quartile of IR	*n*	Detection rates	*P*	OR	95% CI for OR
Hyperglycemia	<1.17^*∗*^	1/104	0.96%	—	—	—
	1.17~	6/106	5.66%	0.094	6.180	(0.731, 52.255)
	1.74~	10/104	9.62%	0.024	10.957	(1.376, 87.232)
	3.28~	12/105	11.43%	0.014	13.290	(1.695, 104.189)
Low HDL-C	<1.17^*∗*^	26/104	25.00%	—	—	—
	1.17~	35/106	33.02%	0.172	1.522	(0.833, 2.778)
	1.74~	39/104	37.50%	0.066	1.746	(0.964, 3.163)
	3.28~	41/105	39.05%	0.031	1.922	(1.063, 3.475)
High TG	<1.17^*∗*^	9/104	8.65%	—	—	—
	1.17~	15/104	14.42%	0.215	1.740	(0.725, 4.174)
	1.74~	17/106	16.04%	0.098	2.063	(0.874, 4.868)
	3.28~	19/105	18.10%	0.034	4.056	(1.258, 8.985)
High LDL-C	<1.17^*∗*^	2/104	1.92%	—	—	—
	1.17~	3/106	2.83%	0.668	1.485	(0.243, 9.077)
	1.74~	3/104	2.88%	0.653	1.515	(0.248, 9.258)
	3.28~	9/105	8.57%	0.040	4.680	(1.646, 16.154)
High TC	<1.17^*∗*^	3/104	2.88%	—	—	—
	1.17~	5/104	4.81%	0.782	1.188	(0.351, 4.020)
	1.74~	6/106	5.66%	0.757	1.212	(0.358, 4.103)
	3.28~	6/105	5.71%	0.475	1.455	(0.858, 7.985)
Abdominal obesity	<1.17^*∗*^	41/104	39.42%	—	—	—
	1.17~	53/104	50.96%	0.095	1.597	(0.921, 2.768)
	1.74~	60/106	56.60%	0.013	2.004	(1.157, 3.473)
	3.28~	65/105	61.90%	0.001	2.497	(1.431, 4.357)

Notes: TG = triglyceride, HDL-C = high-density lipoprotein cholesterol, LDL-C = low-density lipoprotein cholesterol, TC = total cholesterol, IR = insulin resistance, OR = odds ratio, CI = confidence interval, and *∗* = control group.

**Table 3:** The detection rates of glucose and lipid metabolism by IR level in Kazak.

Metabolism	Quartile of IR	*n*	Detection rates	*P*	OR	95% CI for OR
Hyperglycemia	<0.97^*∗*^	8/84	9.52%	—	—	—
	0.97~	10/83	12.05%	0.600	1.301	(0.487, 3.480)
	2.24~	14/83	16.87%	0.166	1.928	(0.762, 4.875)
	5.83~	33/81	40.74%	0.000	6.531	(2.784, 15.323)
Low HDL-C	<0.97^*∗*^	11/83	13.25%	—	—	—
	0.97~	12/83	14.46%	0.822	1.106	(0.458, 2.671)
	2.24~	13/83	15.66%	0.659	1.216	(0.510, 2.895)
	5.83~	21/81	25.93%	0.044	2.291	(1.023, 5.129)
High TG	<0.97^*∗*^	4/83	4.82%	—	—	—
	0.97~	5/83	6.02%	0.732	1.266	(0.328, 4.891)
	2.24~	7/83	8.43%	0.355	1.819	(0.512, 6.466)
	5.83~	16/81	19.75%	0.007	4.862	(1.549, 15.258)
High LDL-C	<0.97^*∗*^	1/83	1.21%	—	—	—
	0.97~	3/83	3.61%	0.500	1.350	(0.293, 6.229)
	2.24~	4/83	4.82%	0.256	2.023	(0.566, 9.812)
	5.83~	7/82	8.54%	0.036	4.569	(1.632, 11.238)
High TC	<0.97^*∗*^	1/83	1.20%	—	—	—
	0.97~	4/83	4.82%	0.517	1.539	(0.418, 5.667)
	2.24~	6/83	7.23%	0.492	1.580	(0.429, 5.821)
	5.83~	6/81	7.41%	0.027	1.862	(1.549, 8.258)
Abdominal obesity	<0.97^*∗*^	46/81	56.79%	—	—	—
	0.97~	53/81	65.43%	0.260	1.440	(0.763, 2.717)
	2.24~	53/79	67.09%	0.181	1.551	(0.815, 2.950)
	5.83~	62/82	75.61%	0.012	2.359	(1.208, 4.604)

Notes: TG = triglyceride, HDL-C = high-density lipoprotein cholesterol, LDL-C = low-density lipoprotein cholesterol, TC = total cholesterol, IR = insulin resistance, OR = odds ratio, CI = confidence interval, and *∗* = control group.

**Table 4:** The detection rates of glucose and lipid metabolism by IR level in Han.

Metabolism	Quartile of IR	*n*	Detection rates	*P*	OR	95% CI for OR
Hyperglycemia	<0.31^*∗*^	7/54	12.96%	—	—	—
	0.31~	8/55	14.55%	0.811	1.075	(0.294, 2.608)
	1.18~	10/57	17.54%	0.754	1.302	(0.392, 3.097)
	2.86~	20/54	37.04%	0.009	3.456	(1.362, 8.769)
Low HDL-C	<0.31^*∗*^	1/55	1.82%	—	—	—
	0.31~	7/57	12.28%	0.043	7.560	(1.098, 63.636)
	1.18~	9/54	16.67%	0.027	10.800	(1.318, 88.506)
	2.86~	9/54	16.67%	0.027	10.800	(1.318, 88.506)
High TG	<0.31^*∗*^	9/55	16.36%	—	—	—
	0.31~	14/54	25.93%	0.225	1.789	(0.700, 4.573)
	1.18~	14/57	24.56%	0.286	1.664	(0.653, 4.238)
	2.86~	21/54	38.89%	0.010	3.253	(1.323, 7.998)
High LDL-C	<0.31^*∗*^	2/55	3.64%	—	—	—
	0.31~	2/54	3.70%	0.985	1.019	(0.138, 7.508)
	1.18~	4/57	7.02%	0.398	2.120	(0.372, 12.088)
	2.86~	5/54	9.26%	0.276	2.548	(0.473, 13.725)
High TC	<0.31^*∗*^	2/55	3.64%	—	—	—
	0.31~	5/54	9.26%	0.247	2.704	(0.501, 14.585)
	1.18~	7/57	12.28%	0.112	3.710	(0.735, 18.715)
	2.86~	8/54	14.81%	0.041	4.829	(1.323, 20.998)
Abdominal obesity	<0.31^*∗*^	26/55	47.27%	—	—	—
	0.31~	35/57	61.40%	0.135	1.774	(0.837, 3.762)
	1.18~	35/54	64.81%	0.067	2.055	(0.952, 4.435)
	2.86~	45/54	83.33%	0.000	5.577	(2.290, 13.583)

Notes: TG = triglyceride, HDL-C = high-density lipoprotein cholesterol, LDL-C = low-density lipoprotein cholesterol, TC = total cholesterol, IR = insulin resistance, OR = odds ratio, CI = confidence interval, and *∗* = control group.

**Table 5:** Multivariate logistic regression analysis of IR with glucose and lipid metabolism in Uygur.

Metabolism	Quartile of IR	*β*	SE	Wald *χ*^2^	*P*	OR	95% CI for OR
Hyperglycemia	<1.17^*∗*^	—	—	—	—	—	—
	1.17~	0.65	0.40	0.53	0.099	1.320	(0.641, 2.599)
	1.74~	1.02	0.52	7.02	0.014	2.432	(1.290, 5.097)
	3.28~	2.12	0.78	12.03	0.001	4.277	(1.702, 9.029)
Low HDL-C	<1.17^*∗*^	—	—	—	—	—	—
	1.17~	0.36	0.42	1.12	0.343	1.560	(1.098, 5.636)
	1.74~	0.47	0.42	1.24	0.227	1.801	(1.018, 7.506)
	3.28~	1.15	0.44	6.04	0.017	2.832	(1.218, 9.506)
High TG	<1.17^*∗*^	—	—	—	—	—	—
	1.17~	0.28	0.35	0.52	0.505	1.289	(0.700, 4.573)
	1.74~	0.77	0.36	4.45	0.046	2.664	(1.253, 4.238)
	3.28~	1.29	0.38	11.88	0.009	3.553	(1.323, 8.798)
High LDL-C	<1.17^*∗*^	—	—	—	—	—	—
	1.17~	0.30	0.38	0.60	0.585	1.019	(0.138, 5.508)
	1.74~	0.35	0.42	3.89	0.068	1.320	(0.772, 6.088)
	3.28~	1.09	0.50	9.79	0.023	2.248	(1.273, 8.725)
Abdominal obesity	<1.17^*∗*^	—	—	—	—	—	—
	1.17~	0.28	0.39	0.59	0.435	1.274	(0.837, 5.762)
	1.74~	1.02	0.42	7.23	0.009	3.055	(1.952, 8.435)
	3.28~	2.88	0.45	20.35	0.000	5.277	(2.390, 11.583)
Smoking	Nonsmoking	—	—	—	—	—	—
	Smoking	0.76	0.29	6.64	0.010	2.13	(1.202, 3.792)

Notes: TG = triglyceride, HDL-C = high-density lipoprotein cholesterol, LDL-C = low-density lipoprotein cholesterol, IR = insulin resistance, OR = odds ratio, CI = confidence interval, and *∗* = control group.

**Table 6:** Multivariate logistic regression analysis of IR with glucose and lipid metabolism in Kazak.

Metabolism	Quartile of IR	*β*	SE	Wald *χ*^2^	*P*	OR	95% CI for OR
Hyperglycemia	<0.97^*∗*^	—	—	—	—	—	—
	0.97~	0.38	0.42	1.23	0.311	1.475	(0.694, 3.608)
	2.24~	1.01	0.66	7.59	0.008	4.302	(1.392, 8.097)
	5.83~	2.88	0.86	40.20	0.001	18.456	(8.362, 44.769)
Low HDL-C	<0.97^*∗*^	—	—	—	—	—	—
	0.97~	0.26	0.32	0.66	0.543	1.260	(0.108, 3.636)
	2.24~	0.32	0.35	1.01	0.427	1.800	(0.318, 5.506)
	5.83~	0.99	0.45	5.64	0.037	2.832	(1.318, 8.526)
High TG	<0.97^*∗*^	—	—	—	—	—	—
	0.97~	0.32	0.40	1.33	0.225	1.889	(0.802, 4.573)
	2.24~	2.12	0.52	8.60	0.026	3.464	(1.643, 6.238)
	5.83~	2.79	0.60	10.08	0.009	4.653	(1.333, 10.998)
High LDL-C	<0.97^*∗*^	—	—	—	—	—	—
	0.97~	0.45	0.42	1.13	0.285	1.571	(0.638, 3.508)
	2.24~	0.47	0.42	1.24	0.271	1.592	(0.672, 3.588)
	5.83~	1.12	0.43	6.64	0.010	3.148	(1.473, 7.725)
High TC	<0.97^*∗*^	—	—	—	—	—	—
	0.97~	0.38	0.40	1.20	0.247	2.704	(0.501, 14.585)
	2.24~	0.42	0.42	1.29	0.112	3.710	(0.735, 18.715)
	5.83~	0.87	0.45	5.59	0.041	4.829	(1.323, 20.998)
Abdominal obesity	<0.97^*∗*^	—	—	—	—	—	—
	0.97~	0.27	0.39	1.12	0.635	1.274	(0.857, 3.562)
	2.24~	0.37	0.40	1.20	0.467	2.055	(0.952, 4.435)
	5.83~	1.02	0.50	7.02	0.001	5.677	(2.380, 13.653)

Notes: TG = triglyceride, HDL-C = high-density lipoprotein cholesterol, LDL-C = low-density lipoprotein cholesterol, TC = total cholesterol, IR = insulin resistance, OR = odds ratio, CI = confidence interval, and *∗* = control group.

**Table 7:** Multivariate logistic regression analysis of IR with glucose and lipid metabolism in Han.

Metabolism	Quartile of IR	*β*	SE	Wald *χ*^2^	*P*	OR	95% CI for OR
Hyperglycemia	<0.31^*∗*^	—	—	—	—	—	—
	0.31~	0.29	0.35	0.55	0.511	1.275	(0.594, 2.608)
	1.18~	0.92	0.37	5.59	0.044	2.302	(1.192, 5.097)
	2.86~	1.33	0.40	12.65	0.001	3.456	(1.362, 8.769)
Low HDL-C	<0.31^*∗*^	—	—	—	—	—	—
	0.31~	0.78	0.50	2.56	0.083	6.560	(2.098, 59.636)
	1.18~	1.23	0.55	8.52	0.037	11.023	(1.318, 80.506)
	2.86~	2.77	0.60	36.20	0.001	17.156	(2.318, 91.506)
High TG	<0.31^*∗*^	—	—	—	—	—	—
	0.31~	0.31	0.42	0.66	0.225	1.789	(0.700, 4.573)
	1.18~	0.35	0.44	0.56	0.286	1.664	(0.653, 4.238)
	2.86~	1.03	0.50	3.56	0.020	4.253	(1.323, 8.998)
High TC	<0.31^*∗*^	—	—	—	—	—	—
	0.31~	0.28	0.31	0.48	0.247	2.704	(0.501, 14.585)
	1.18~	0.32	0.39	0.99	0.112	3.710	(0.735, 18.715)
	2.86~	0.38	0.42	2.89	0.041	4.829	(1.323, 20.998)
Abdominal obesity	<0.31^*∗*^	—	—	—	—	—	—
	0.31~	0.25	0.35	0.51	0.435	1.274	(0.637, 2.762)
	1.18~	0.92	0.36	6.44	0.012	2.455	(1.252, 4.435)
	2.86~	1.27	0.37	11.75	0.001	3.577	(1.690, 7.583)
Smoking	Nonsmoking	—	—	—	—	—	—
	Smoking	1.22	0.34	13.08	0.001	3.39	(1.752, 6.592)

Notes: TG = triglyceride, HDL-C = high-density lipoprotein cholesterol, TC = total cholesterol, IR = insulin resistance, OR = odds ratio, CI = confidence interval, and *∗* = control group.

## Abbreviations

BP: Blood pressure
CI: Confidence interval
FBG: Fasting blood glucose
HDL-C: High-density lipoprotein cholesterol
HOMA-IR: Homeostasis model assessment of insulin resistance
IR: Insulin resistance
LDL-C: Low-density lipoprotein cholesterol
MS: Metabolic syndrome
OR: Odds ratio
TC: Total cholesterol
TG: Triglyceride
WC: Waist circumference
FINS: Fasting serum insulin.
